# SCI NutriTool: Development and Validation of a Questionnaire to Assess Non-Adherence to the Healthy Food Pyramid in Individuals with Spinal Cord Injury in Switzerland

**DOI:** 10.3390/nu18111737

**Published:** 2026-05-28

**Authors:** Marija Glisic, Inge Eriks-Hoogland, Angeline Chatelan, Khadija Maham, Silvia Mattmann, Pedro Marques-Vidal, Sara Rubinelli, Claudio Perret

**Affiliations:** 1 Swiss Paraplegic Research, 6207 Nottwil, Switzerland; inge.eriks@paraplegie.ch (I.E.-H.); khadija.maham@stud.unilu.ch (K.M.); claudio.perret@paraplegie.ch (C.P.); 2 Institute of Social and Preventive Medicine (ISPM), University of Bern, 3012 Bern, Switzerland; 3 Faculty of Health Sciences and Medicine, University of Lucerne, 6005 Lucerne, Switzerland; sara.rubinelli@unilu.ch; 4 Swiss Paraplegic Centre, 6207 Nottwil, Switzerland; silvia.mattmann@paraplegie.ch; 5 Department of Nutrition and Dietetics, Geneva School of Health Sciences, HES-SO University of Applied Sciences and Arts Western Switzerland, 1227 Geneva, Switzerland; angeline.chatelan@hesge.ch; 6 Department of Medicine, Internal Medicine, Lausanne University Hospital (CHUV) and University of Lausanne, 1011 Lausanne, Switzerland

**Keywords:** spinal cord injury, diet, food pyramid, questionnaire validation

## Abstract

Background/Objective: Rapid, validated dietary screening tools are lacking for individuals with spinal cord injury (SCI), where routine clinical check-ups do not allow sufficient time for extensive dietary assessments typically required to evaluate adherence to dietary recommendations. We developed a 15-item dietary screener (SCI NutriTool) and evaluated its accuracy in classifying non-adherence to a healthy food pyramid compared with a validated food frequency questionnaire (FFQ). Methods: The SCI NutriTool was developed through literature review and expert consensus. In a validation study, 51 adults with SCI (mean age 57.0 years; 76.5% men; 68.8% traumatic injury) completed the SCI NutriTool twice and a validated 97-item FFQ, which served as the reference method. Results: The SCI NutriTool demonstrated substantial variability in performance across food groups, reflecting its domain-specific screening properties. Sensitivity was high for fruits and vegetables (91.7%), protein-rich foods (90.5%), and sweetened/alcoholic beverages and snacks (82.4%), with relatively high positive predictive values (PPV: 73.7–90.5%), supporting the tool’s ability to identify individuals who are likely non-adherent and may benefit from further nutritional assessment or counselling. In contrast, for starchy foods and nuts, oils, and fatty spreads/sauces, sensitivity was low (20.0% and 50.0%), while specificity was modest. This indicates that the tool performs better in correctly identifying adherent individuals in these domains, which is reflected in higher negative predictive values (NPV: up to 94.1%). However, the low sensitivity suggests that individuals with non-adherence may be missed, limiting the tool’s usefulness as an early screening trigger for these food groups. Conclusions: The SCI NutriTool’s performance varies across food groups, demonstrating a stronger ability to identify non-adherence in protein-rich foods, fruit and vegetables, sweetened and alcoholic beverages, and snacks, but limited discriminatory capacity for others. In particular, it is not suitable for screening non-adherence to starchy foods and fats. Accordingly, it is best used as a triage tool to guide further dietary assessment and targeted nutritional interventions rather than as a standalone diagnostic instrument.

## 1. Introduction

Individuals living with spinal cord injury (SCI) undergo extensive pathophysiological changes that predispose them to a range of secondary health complications [1,2,3]. In addition to the well-documented increased risk of metabolic disorders (e.g., obesity, dyslipidemia, insulin resistance) and cardiovascular diseases, individuals with SCI frequently experience chronic low-grade systemic inflammation, elevated oxidative stress, and impaired immune response [4,5,6,7,8]. These pathophysiological changes contribute to higher susceptibility to pressure injuries and recurrent infections [5,7]. These secondary health complications pose a substantial burden on quality of life, increase healthcare utilization, and exacerbate long-term morbidity and mortality [9,10,11,12,13,14].

Diet represents a central and modifiable determinant of many physiological and pathological processes involved in health complications following SCI. Dietary patterns influence systemic inflammation and oxidative stress through nutrient intake, diet quality and balance, thereby affecting metabolic health, immune responses, gut function, and tissue integrity [15]. In individuals with SCI, who typically experience reduced energy expenditure, altered body composition, and autonomic regulation (involuntary control of functions like heart rate and digestion), suboptimal dietary intake may further exacerbate inflammatory pathways and contribute to the development or progression of secondary health conditions [16,17,18]. Adherence to food-based dietary guidelines (FBDG) has the potential to mitigate inflammation, support metabolic and gastrointestinal health, and promote overall well-being in this population [19]. The majority of individuals with SCI may not appropriately follow dietary recommendations after the injury [20,21,22,23,24]. Recent findings demonstrated greater energy intake relative to energy requirements in individuals with chronic SCI, and an imbalance in intake of fiber, macronutrients (excessive protein and carbohydrate intake) and micronutrients (low intake of vitamins A, B5, B7, B9, D, and E, and potassium and calcium) compared to dietary guidelines for Americans [25].

In contrast, routine dietary assessment in individuals with SCI remains challenging in both clinical practice and research [26]. Comprehensive methods such as food frequency questionnaires (FFQs), multiple 24 h recalls, or food records are resource-intensive, time-consuming, and require trained personnel [27]. These limitations reduce feasibility in busy clinical settings and large-scale studies, where rapid identification of dietary risk is often needed to guide clinical decision-making. As a result, nutrition-related risk factors may remain unrecognized and unaddressed.

Brief dietary screening tools offer a pragmatic alternative to detailed dietary assessment methods by enabling rapid classification of adherence to dietary recommendations. A range of such instruments has been developed, predominantly targeting general populations; however, these tools are typically limited in scope [28,29], often covering only selected food groups [30,31,32,33,34] or single dietary constructs [35,36,37], and have rarely been validated for clinical populations with specific nutritional risk profiles. Individuals with SCI present distinct metabolic, functional, and lifestyle-related dietary challenges that may not be adequately captured by generic screening instruments. In addition, existing brief dietary screeners have limited evidence of cross-cultural adaptation and are predominantly available in English, further restricting their applicability in multilingual healthcare settings such as Switzerland. To date, there is a lack of rapid, validated dietary screening tools specifically tailored to adults with SCI and aligned with food-based dietary guidelines relevant to nutrition care and counselling in this population.

To address this gap, we developed the SCI NutriTool, a short 15-item dietary instrument designed to rapidly assess non-adherence to the national FBDG (Swiss Food Pyramid) in adults with SCI. The objective of this study was to evaluate the accuracy of the SCI NutriTool in classifying non-adherence across key food groups, using a validated FFQ as the reference method, and to assess test–retest reliability. By providing a feasible first-line screening instrument, the SCI NutriTool aims to support more detailed nutritional assessment and personalized interventions in SCI care.

## 2. Materials and Methods

### 2.1. Study Setting and Population

All male and female individuals with chronic (≥1 year since injury) traumatic and non-traumatic SCI aged ≥18 years were eligible to participate. Convenience sampling was conducted among individuals visiting the Swiss Paraplegic Centre in Nottwil, Switzerland, for annual health check-up and through local SCI associations. Individuals with congenital conditions leading to para- or tetraplegia including spina bifida, neurodegenerative diseases such as multiple sclerosis, Guillain-Barré Syndrome or Amyotrophic Lateral Sclerosis and newly diagnosed SCI as part of palliative “end-of-life-care” treatment (e.g., tumor disease with metastases) were not eligible to participate. Formal consent was obtained before the participant was given any study questionnaire. All participants had at least 24 h to consider their participation.

### 2.2. Study Procedures

Individuals were recruited between March 2024 and April 2025. The study involved two dietary questionnaires (one long and one short) that collected information on dietary intake over the past month. The short questionnaire was repeated after two weeks to assess test–retest reliability. A further questionnaire on physical activity was administered at baseline. A research assistant collected relevant clinical information from the electronic medical records of the study participants (i.e., parameters routinely measured during the annual check-up).

Participants could choose to complete the questionnaires either on paper or via our web-based platform. The web platform (https://sci-nutritool.ch/) was developed specifically for the current study and was accessible only to individuals who consented to participate. Participants who chose to complete the questionnaires online received login details from the study personnel. If a participant opted to complete the paper-based questionnaires, a stamped return envelope was provided by the study personnel.

### 2.3. Short Diet Quality Screener/SCI NutriTool

The short dietary assessment questionnaire, i.e., SCI NutriTool, was developed through a multidisciplinary and iterative process involving a research team composed of epidemiologists with expertise in cardiometabolic health and nutrition, clinicians specialized in SCI, a nutritionist, a health science specialist, a data scientist, a web developer, and a communication specialist. At this stage, individuals with SCI were not directly involved, as the primary focus was to ensure the development of methodologically robust questionnaire content, aligned with the best available evidence and existing validated dietary assessment instruments, and informed by expert consensus. Future phases will incorporate user perspectives to further refine tool’s acceptability, usability and relevance. The development process was informed by multiple complementary sources. First, a systematic review of the literature was conducted to identify dietary patterns and food groups associated with health benefits in individuals with SCI, thereby establishing the scientific basis for relevant nutritional components [19]. Second, SCI dietary recommendations [38] and national FBDG (Swiss Food Pyramid) [39] were reviewed to ensure alignment with country-specific nutritional recommendations and cultural context. To enhance methodological rigor and comparability with established dietary assessment approaches, items from a validated Swiss long food frequency questionnaire were examined and used as a reference for question structure and food categorization [40]. In addition, existing dietary screening tools and frameworks available through www.nutritools.org (accessed on 20 September 2021) were reviewed to inform best practices in questionnaire design. Based on this evidence base, an initial pool of items was generated and iteratively refined through team discussions.

**Figure 1:**
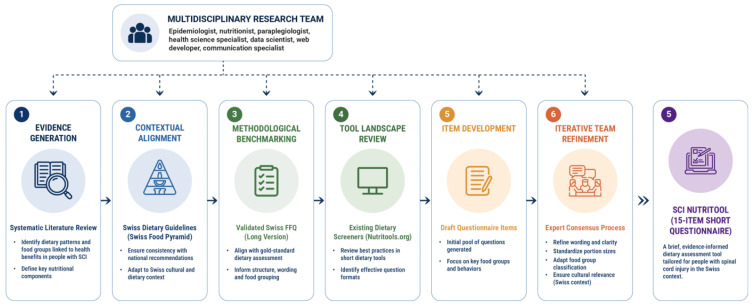
SCI NutriTool Development Process.

This process (Figure 1) resulted in a final set of 15 questions, with careful consideration given to portion sizes, food group classification, clarity of language, and cultural appropriateness within the Swiss context.

Study participants were asked to base their responses on their usual dietary behaviors over the previous four weeks, reporting their intake of 15 food items grouped into five food categories. Daily or weekly food frequency consumption was arranged into five response categories: (i) “none” or “never”; (ii) “once a day”; (iii) “twice a day”; (iv) “three times a day”; and (v) “more than three times a day.” The first food group category referred to protein-rich foods and included four items: (i) red meat and processed meat products; (ii) other types of animal-based protein-rich foods (poultry, fish, seafood, eggs); (iii) plant-based protein-rich foods (tofu, tempeh, or soy); and (iv) milk and dairy products. The second category included two items: (i) fruit and (ii) vegetable intake, in the raw, cooked, or dried form, but excluding juices. The third category was a single-item category that assessed the intake of starchy foods, such as pasta, potatoes, polenta, oatmeal, couscous, other grains, or legumes. The fourth category referred to nuts, oils and fatty spreads/sauces and consisted of four items: (i) unsalted nuts, kernels, or seeds (e.g., almonds, sunflower seeds, sesame seeds); (ii) oil or butter used for cooking, frying, or salad dressing; (iii) cream and fatty sauces (e.g., pesto, cream-based sauces) used for cooking, salad, or food dressing; and (iv) butter, margarine, or mayonnaise used as a spread. The fifth category referred to drinking habits, snacks, and dessert intake and consisted of four items: (i) sugar-sweetened beverages; (ii) alcoholic beverages; (iii) sweets or desserts; and (iv) salty snacks or high-fat dishes. The short questionnaire is provided as a separate document.

### 2.4. Food Frequency Questionnaire

Dietary intake over the four weeks prior to the interview was assessed using a self-administered, semi-quantitative FFQ that included portion sizes [40]. This FFQ was validated in the Geneva population but is also applicable to German- and Italian-speaking Swiss populations, as most foods included are commonly available across Switzerland. The FFQ comprised 97 food items, covering over 90% of caloric, protein, fat, carbohydrate, alcohol, cholesterol, vitamin D, and retinol intake, and approximately 85% of fiber, carotene, and iron intake. For each item, participants reported consumption frequency, ranging from “less than once during the last four weeks” to “two or more times per day,” and indicated their average serving size relative to a reference portion (smaller, equal, or larger). To harmonize responses from the long FFQ and make them comparable with the SCI NutriTool, we grouped food items into the five predefined categories used in the short questionnaire and converted frequency data into five standardized response categories: (i) “none” or “never”; (ii) “once a day”; (iii) “two times a day”; (iv) “three times a day”; and (v) “more than three times a day.” Since respondents could report their intake using monthly, weekly, or daily frequencies, reported intake (ranging from “never in the last 4 weeks” to “two or more times per day”) was converted into the average number of servings per day and per week using predefined conversion factors. Conversion factors for monthly and weekly categories were calculated as the midpoint of the reported range divided by the relevant period (e.g., 2–3×/month → 2.5 ÷ 30 days ≈ 0.083 servings/day). Daily categories were converted directly to their reported frequency, and the highest frequency category (“two or more times per day”) was assigned a conservative value of 2 servings/day. Weekly servings were derived by multiplying daily equivalents by seven (e.g., 1×/day = 7 servings/week). This approach enabled consistent quantification of dietary intake across participants while preserving the relative differences between FFQ response categories, supporting subsequent analyses.

### 2.5. Non-Adherence to Swiss Food Pyramid 

We defined dietary recommendation for individuals with SCI for this study using the national FBDG, i.e., the Swiss Food Pyramid from the Swiss Federal Food Safety and Veterinary Office [39] and findings from our systematic review identifying the most promising dietary patterns associated with health benefits in SCI [19] and existing SCI-specific dietary recommendations [38].

Overall, our proposed recommendations were largely consistent with the Swiss FBDG for the general population. Specifically, an acceptable level of adherence included: (i) a daily intake of at least three portions of vegetables and two portions of fruits; (ii) up to three daily portions of whole grains or other starchy foods; and (iii) one serving of unsalted, unprocessed nuts or seeds per day. In addition, no more than three servings of plant oils or butter for food preparation were recommended, along with up to one serving per day of fatty spreads/sauces. Due to the specific considerations related to gastrointestinal function, bowel management and microbiome diversity in individuals with SCI [41], we slightly modified the recommendations regarding red and processed meat consumption. Acceptable adherence to protein-rich foods was defined as no more than two servings of red and processed meat per week, together with one daily portion of protein-rich foods (animal- or plant-based) and two to three portions of dairy products per day. Further, acceptable intake of alcoholic and sweetened beverages, sweets and salty snacks was defined as consuming these items only in small quantities. Participants were classified as non-adherent unless they met all the following criteria: alcohol consumption within acceptable limits of ≤3 servings per week and a combined intake of sugar-sweetened beverages, sweets/desserts, and salty/fatty snacks below the defined threshold of 6 servings per week. Our recommendations for this food group are more conservative than the Swiss FBDG recommendations since individuals with SCI often experience a sustained imbalance between energy intake and energy expenditure. Evidence shows that daily caloric intake exceeds metabolic needs by approximately 300–500 kcal, with pooled estimates indicating a resting metabolic rate of about 1492 kcal/day compared to an intake of 1876 kcal/day [25,42,43]. Although this excess may appear modest, over time it contributes to the accumulation of body fat and increases the risk of adverse cardiometabolic outcomes, including dyslipidemia, impaired glucose tolerance, insulin resistance, hypertension, and systemic inflammation [44]. Given this increased risk, stricter limitations on energy-dense, nutrient-poor foods (such as alcohol, sugar-sweetened beverages, sweets, and salty snacks) are warranted to help prevent chronic positive energy balance and support long-term cardiometabolic health.

### 2.6. Other Variables

Basic demographic information of individuals with SCI (e.g., age, sex), injury characteristics (e.g., injury duration, level and completeness), and accompanying medical conditions (i.e., prevalent CVDs, type 2 diabetes and obesity) were extracted from patient medical records. Physical activity was assessed using the Physical Activity Scale for Individuals with Physical Disabilities and was administered during an interview conducted by a research assistant [22]. The number of minutes per week of leisure time physical activity performed at each intensity (mild, moderate and heavy) was calculated by multiplying the days of activity by the minutes of activity. The definition of sufficient physical activity was based on the guideline by Ginis et al., suggesting that cardiometabolic health benefits can be achieved through a minimum of 30 min of moderate-to-vigorous aerobic exercise performed three times weekly [45].

### 2.7. Statistical Analyses 

Descriptive statistics were used to summarize personal and clinical characteristics of the study participants. Adherence to the established dietary recommendations (as described above) was assessed using the long FFQ which served as the reference instrument. Fisher’s exact test was used to compare the proportion of participants who were non-adherent to food group recommendations across sex, injury level, obesity status, and physical activity status. Adherence was operationalized for each food group and based on the intake frequency and was further dichotomized according to established dietary recommendations. Incomplete responses in the FFQ resulted in missing data for certain food groups, which limited the ability to determine adherence for those specific groups. To maximize sample size, analyses were therefore conducted using all available (complete) data for each individual food group. Consequently, the sample size varied across food groups, ranging from 25 to 35 participants in the main analyses. Criterion validity of the SCI NutriTool was evaluated against the FFQ by harmonizing food items from both instruments into the same five food groups and applying identical guideline-based cut-offs. The ability of the SCI NutriTool to correctly classify non-adherence was assessed by calculating sensitivity and specificity. Positive and negative predictive values (PPV and NPV) were calculated to explore the clinical utility of a screening test by measuring the probability that a person truly does not adhere (PPV) or does adhere (NPV) to dietary recommendations based on the short questionnaire responses. Two-week test–retest was assessed using percent agreement and Cohen’s Kappa. Prevalence-Adjusted, Bias-Adjusted Kappa (PABAK) was used alongside Cohen’s kappa to identify if low agreement was due to a genuine lack of consistency or just imbalanced data. The value of Kappa, identifying the strength of agreement, was categorized according to Masson et al. as follows: <0.20: poor, 0.21–0.40: fair, 0.41–0.60: moderate, 0.61–0.80: good, 0.81–1.00: very good [46]. To explore the possibility of selection bias, we compared the basic characteristics of individuals with complete data on both the SCI NutriTool and FFQ versus those who had missing information as a sensitivity analysis. Statistical analyses were conducted in Stata 18 (Stata Corp, College Station, TX, USA).

### 2.8. Language Editing Using Artificial Intelligence

To enhance linguistic clarity and grammatical accuracy, we used ChatGPT (OpenAI, San Francisco, CA, USA; GPT-5.3-mini, accessed via the ChatGPT interface). The tool was used solely for language editing across the manuscript, including sentence-level improvements in grammar, syntax, punctuation, and readability. It was not used to generate or modify scientific content, data interpretation, or intellectual contributions. All AI-assisted revisions were carefully reviewed by humans to ensure that the scientific meaning and integrity of the text were fully preserved.

## 3. Results

### 3.1. Study Population Selection and Characteristics 

**Figure 2:**
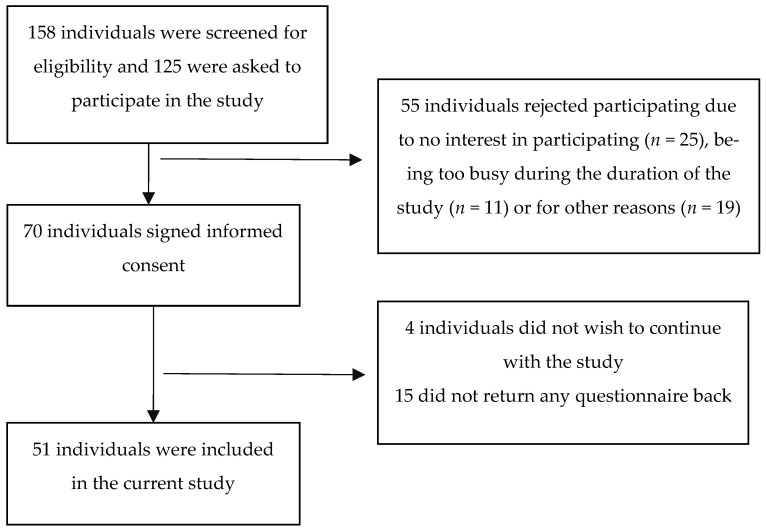
Flowchart of study participants.

A total of 125 individuals were invited to participate in the study, of whom fifty-five declined participation, due to lack of interest, being too busy during the study period, or other reasons. Seventy participants provided informed consent. During the study, four participants withdrew, and 15 did not return any questionnaires. The analysis included 51 participants (Figure 2).

The mean age of study participants was 57.0 years (SD 16.6), of whom 39 (76.5%) were male. Most (68.6%) had a traumatic spinal cord injury, thoracic or lumbo-sacral lesions (60.7%) and a motor complete injury (41.2%). The mean body mass index (BMI) was 25.2 kg/m^2^ (SD 5.8), and 23.5% were classified as obese. Only one quarter of participants (26.7%) complied with physical activity recommendations (Table 1).

### 3.2. Non-Adherence to the Established Dietary Recommendations

Based on data collected using the FFQ (reference questionnaire), non-adherence to dietary recommendations varied substantially across food group categories (Table 2). The lowest adherence was observed for protein-rich foods and for fruits and vegetables, with 81.5% and 72.7% of participants not meeting the respective guidelines. Approximately 48% of participants did not adhere to recommendations for sweetened and alcoholic beverages, as well as for sweet and salty snacks. In contrast, adherence was high for nuts, oils, and fatty spreads or sauces (94%) and for starchy foods (85%).

Males were more likely to report excessive consumption of alcohol, sugar-sweetened beverages, and snacks, and were also more likely to report suboptimal intake of fruits and vegetables. No sex differences were observed for the intake of starchy foods or nuts, oils and fatty spreads/sauces. Furthermore, adherence did not differ according to obesity, injury level or level of physical activity (Table 3).

### 3.3. SCI NutriTool Performance

The SCI NutriTool showed variable sensitivity and specificity across food groups compared with the FFQ (Table 2), indicating heterogeneous screening performance depending on the dietary domain assessed. For sweetened and alcoholic beverages, and sweet and salty snacks, performance was relatively balanced, with acceptable sensitivity (82.4%) and specificity (72.2%), a positive predictive value (PPV) of 73.7%, and a negative predictive value (NPV) of 81.3%. This suggests that the SCI NutriTool can effectively flag individuals likely to be non-adherent while reasonably identifying those who are adherent to recommendations in these domains.

For protein-rich foods, sensitivity (90.5%) and PPV (90.5%) were high, while sensitivity and NPV were 50%. This indicates that the tool is effective at flagging individuals who are likely non-adherent in this domain, although some adherent individuals may also be flagged for further dietary assessment. A similar pattern was observed for fruit and vegetable intake, with very high sensitivity (91.7%) but low specificity (22.2%). Given the high pre-test probability of non-adherence (72.7%), the PPV remained relatively high (75.9%), indicating that individuals flagged as non-adherent were likely to truly be non-adherent. However, the low NPV (50%) shows that the classification of adherence may not be reliable. In this context, the SCI NutriTool is better suited to flagging individuals who may need additional nutritional counselling than to confirming adherence.

In contrast, starchy foods showed low sensitivity (20.0%) but high specificity (96.4%), with a PPV of 50.0% and an NPV of 87.1%. This pattern suggests that the tool was more capable of identifying adherent individuals in this domain than of detecting non-adherence, which limits its usefulness as a self-screening instrument for early identification of suboptimal intake. A similar imbalance was observed for nuts, oils, and fatty spreads or sauces, where sensitivity was very low (6.7%) despite relatively high specificity (94.1%). Although the NPV was high (94.1%), this likely reflects the underlying distribution of adherence rather than strong discriminative performance; therefore, reassurance from a negative result should be interpreted cautiously.

### 3.4. Test–Retest Reliability 

Test–retest reliability of the SCI NutriTool showed moderate to good agreement across food group categories (Table 4). The agreement ranged from 74% to 83%. PABAK values suggested more consistent reliability across food groups (0.48–0.66), corresponding to moderate to good agreement. Overall, the SCI NutriTool demonstrated acceptable test–retest reliability, with the strongest stability observed for starchy foods and sweetened, alcoholic beverages and salty snacks and comparatively lower reliability for other food group categories. 

## 4. Discussion

In the current study, we observed substantial heterogeneity in dietary behaviors, with particularly low adherence to protein-rich food recommendations and fruit and vegetable intake, and only moderate compliance with alcoholic and sweetened beverage and salty and sweet snack intake, underscoring persistent gaps in dietary quality within this high-risk group [24,38,47]. Compared with the FFQ, the SCI NutriTool showed domain-specific performance as a first-line self-screening and triage instrument rather than a diagnostic tool. 

The SCI NutriTool performed best in domains where sensitivity (with acceptable 95% CIs widths) and PPV were relatively high, including protein-rich foods and fruit and vegetables, suggesting that it can effectively flag individuals who may be non-adherent and who may benefit from further dietary assessment or nutritional counselling. Performance was weak for starchy foods and nuts, oils, and fatty spreads, where low sensitivity suggests that the tool may miss individuals with suboptimal intake. Although negative predictive values were relatively high in these domains, they should be interpreted cautiously because they are likely influenced by the underlying prevalence of adherence rather than by strong discriminatory ability. These findings indicate that the SCI NutriTool may not be adequate even for screening purposes in starchy foods and nuts, oils, and fatty spreads.

The most balanced performance was observed for sweetened beverages, alcoholic beverages, and salty snacks, where sensitivity and specificity were both acceptable. This suggests that the tool is particularly useful for identifying high-risk discretionary consumption patterns.

Overall, the primary value of SCI NutriTool lies in rapidly flagging individuals with probable dietary non-adherence who may benefit from targeted, more detailed dietary assessments and prioritized nutritional counselling by specialized professionals, while still ensuring that all patients receive baseline advice on healthy eating. 

### 4.1. Limitations of Current Study 

Several methodological considerations warrant careful discussion to contextualize the interpretation of our findings. 

First, the observed heterogeneity in sensitivity and specificity across food group categories likely reflects several methodological and behavioral factors. First, differences in cognitive burden associated with recalling and estimating intake may contribute to variable classification accuracy. Foods that are salient and typically consumed as discrete items, such as fruits, vegetables, and sweetened beverages, are generally easier to conceptualize and report, potentially enhancing the detection of non-adherence. In contrast, items such as nuts, oils, and fatty spreads/oils and starchy foods are often incorporated into mixed dishes, consumed irregularly, or eaten in small quantities, which increases the likelihood of misclassification. Further, the FFQ used as the reference method in the current study does not comprehensively capture certain food groups included in our adherence definition, particularly pulses and pulse-based products (e.g., tofu), nuts, and seeds [40]. These foods represent important sources of plant-based fats and are partially captured within the broader “fatty foods” or protein-rich food categories in the scoring algorithm. Their omission or limited representation in the FFQ may therefore lead to an underestimation of total intake of these foods when using the reference method. Consequently, participants who consumed these items in accordance with the recommendations may have been incorrectly classified as non-adherent in the FFQ-based assessment. This discrepancy between the two instruments could reduce the sensitivity of the short dietary assessment tool for identifying individuals who truly did not meet the recommended intake of nuts, oils, and fatty spreads/sauces.

Second, the precision of the estimated screening performance was limited, as several sensitivity and specificity estimates were accompanied by wide 95% confidence intervals, reflecting the relatively small number of observations within individual food groups. A formal sample size calculation was not feasible for this validation study due to the lack of prior information on the prevalence of non-adherence across specific food groups. Consequently, the study may have been underpowered to generate precise estimates, particularly for domains with low prevalence or few misclassified participants. As a result, the reported performance measures should be interpreted with caution, especially for food groups with wide confidence intervals, including starchy foods and nuts, oils, and fatty spreads/sauces. The number of participants with complete information for both the NutriTool and FFQ varied across food groups (ranging from 25 for protein-rich foods to 35 for sweetened, alcoholic beverages, and salty snacks). We compared age, sex, injury characteristics, obesity status, and adherence to physical activity guidelines between participants with complete versus incomplete data and found no statistically significant differences, suggesting that missingness was unlikely to introduce systematic bias. In addition, the use of a convenience sample from a specialized Swiss SCI setting may limit the external validity of the findings [48]. Participants were recruited during routine annual check-ups, meaning that individuals who did not attend, potentially due to acute health complications or hospitalization, were not captured. As a result, the sample may overrepresent individuals who are more regularly engaged in care and in relatively stable health, thereby limiting the generalizability of the findings to the broader SCI population. Further, Switzerland is characterized by marked linguistic and cultural diversity, which may influence dietary habits, food terminology, and interpretation of questionnaire items, even when using a German-language version of the tool. Although the SCI NutriTool was developed and administered in German, its applicability to French- and Italian-speaking regions, as well as to individuals from different cultural and dietary backgrounds, requires further investigation. Therefore, while the present study provides important preliminary evidence for the performance of the SCI NutriTool, further validation in larger, multi-center, and multilingual SCI populations is needed to confirm its generalizability and cross-cultural applicability.

Third, the operational definition of adherence and the distribution of intake within the study population may influence diagnostic performance. When a substantial proportion of participants cluster around predefined cut-off values, small reporting errors can lead to disproportionate shifts in classification, thereby affecting sensitivity and specificity. In addition, prevalence-dependent effects may occur when adherence is either uncommon or highly prevalent within specific food groups, resulting in imbalanced performance metrics [49].

Fourth, a key limitation of this study relates to the choice of reference method. Although a 4-day food diary was originally planned as the reference method due to its closer approximation of actual intake and reduced reliance on memory, completion rates were insufficient, with only 21 individuals (41%) providing somewhat usable data despite reminders and support. As a result, we used the FFQ, which was available for almost all participants who agreed to take part in the study. FFQs are, however, subject to both systematic and random measurement error, including recall bias, as participants are required to summarize their usual dietary intake over an extended period (e.g., the past month or longer) [50]. This may lead to imprecision in estimating frequency and portion sizes, as well as systematic overreporting of socially desirable foods (e.g., fruits and vegetables) and underreporting of less desirable items (e.g., alcohol or salty snacks). Such patterns may result in differential misclassification across food groups. For example, overreporting of healthy foods may lead to an underestimation of non-adherence in the reference method, which could in turn lower the estimated sensitivity of the SCI NutriTool while inflating specificity. In contrast, random measurement error is likely to attenuate associations, resulting in more conservative estimates of agreement. However, the extent to which misclassification is differential or non-differential in this study cannot be determined, as we are unable to assess whether reporting errors differ between adherent and non-adherent individuals. At the same time, it is important to note that the SCI NutriTool also assesses habitual intake over a similar recall period (past month), which provides some conceptual alignment between the index and reference measures in terms of temporal framework. In contrast, the originally planned food diary captures short-term intake over four days and may be more sensitive to day-to-day variability, which is not necessarily representative of habitual dietary patterns. Nevertheless, the use of an FFQ as the reference standard likely introduced both attenuation of agreement and potential bias in sensitivity and specificity estimates. Therefore, the reported screening performance should be interpreted as reflecting limitations of both instruments, rather than the SCI NutriTool alone.

Finally, the inherent simplification of brief screening tools may differentially affect food groups characterized by complex consumption patterns. Collapsing diverse dietary sources into a limited number of items may be sufficient for foods with straightforward intake patterns but less adequate for those derived from multiple ingredients and preparation methods. Together, these factors likely explain the observed variation in the tool’s capacity to correctly classify adherence across dietary domains.

### 4.2. Outlook: Personalized Diet Adherence Reporting System

**Figure 3:**
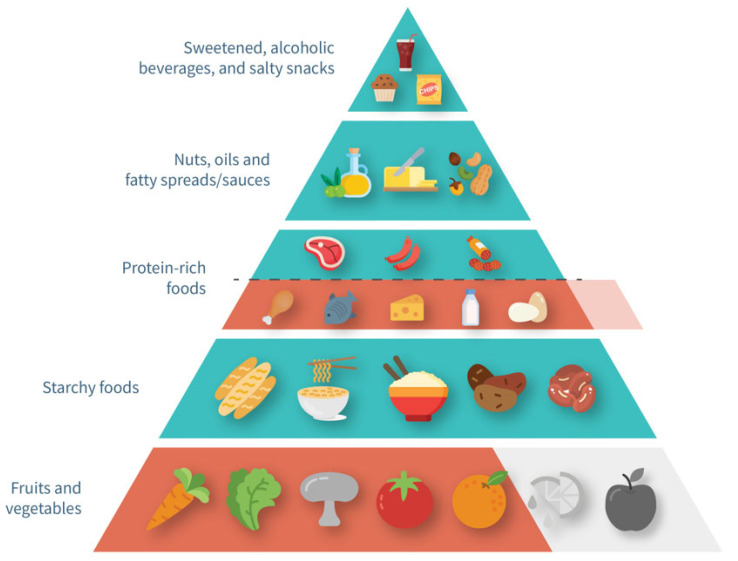
Personalized dietary reported based on SCI NutriTool responses. The orange color indicates non-adherence to the recommended intake for a given food group, reflecting either excessive or insufficient consumption, whereas the green color indicates adherence to the recommended intake for that food group. The dashed line separates red and processed meat (a category with established evidence of health harm) from other protein-rich food sources.

Building on the SCI NutriTool, we developed a personalized diet adherence reporting system (Figure 3), which will be integrated into the online platform during the implementation phase. The system generates an individualized diet quality report based on responses to the 15-item questionnaire. In designing the tool, we drew inspiration from existing food pyramids and dietary guidelines in the literature, including the Swiss Food Pyramid, the Mediterranean Diet Pyramid, and international models, to ensure that recommendations align with widely recognized dietary principles and promote balanced dietary patterns.

The report adopts a food pyramid structure to visually represent alignment with dietary recommendations across five key food groups: fruits and vegetables; starchy foods and legumes; protein-rich foods; nuts, seeds, and healthy fats; and beverages and snacks/desserts. Each food group is color-coded, with visual indicators denoting adherence or areas requiring improvement. This visual and intuitive reporting format is designed to facilitate user engagement and to support informed dietary decision-making. Importantly, the pyramid-based structure provides flexibility, allowing the reporting system to be readily updated if dietary recommendations evolve in the future, while maintaining the same intuitive visualization framework.

Future refinements will focus on improving linguistic accessibility and enhancing the clinical relevance and personalization of feedback. Given Switzerland’s multilingual context, future work should explore translation and cultural adaptation of the SCI NutriTool into French and Italian, in addition to evaluating whether Swiss German linguistic variations influence item interpretation and response patterns. This will be particularly important to ensure cross-regional applicability and reduce potential measurement bias related to language and cultural dietary differences. Although individuals with SCI were not directly involved in the initial development of the short questionnaire, this was because the primary aim at this stage was to develop a valid and methodologically robust tool. In future adaptation work, both individuals with SCI and health professionals should be involved to further refine the tool and enhance its clinical relevance.

In parallel, further development should address the tailoring of feedback algorithms to relevant clinical characteristics in SCI, such as injury level, metabolic risk profile, and comorbid conditions. This would allow more individualized interpretation of dietary risk patterns rather than a one-size-fits-all classification approach. In future pilot implementation studies, the feasibility of integrating the SCI NutriTool into structured clinical workflows could be tested, including its use as a trigger for referral to nutrition professionals for individuals identified as potentially non-adherent.

Finally, future research should focus on evaluating the tool’s implementation in real-world clinical practice, including its acceptability to patients and healthcare providers, its impact on referral rates to dietetic services, and its effectiveness in improving dietary behaviors and health outcomes when embedded within multidisciplinary care models. Additionally, further validation in larger, more diverse cohorts is needed to refine domain-specific thresholds and to explore whether tailored scoring algorithms or adaptive screening strategies could enhance performance in low-sensitivity domains. Integration of the SCI NutriTool into digital health platforms, such as electronic health records or mobile health applications, could facilitate automated flagging of at-risk individuals and streamline pathways to nutritional counselling.

## 5. Conclusions

The SCI NutriTool is not intended to diagnose dietary inadequacy or confirm adherence; rather, it is a self-screening instrument designed to identify individuals with SCI who may benefit from further dietary assessment and nutritional counselling. It demonstrates acceptable test–retest reliability and domain-specific screening performance, which is strongest for protein-rich foods, fruits and vegetables, and items such as sweetened beverages and snacks. However, it may not be suitable for detecting non-adherence in domains related to starchy foods and fats, oils, and spreads.

Overall, the SCI NutriTool may support the early identification of nutritional risk and facilitate timely access to specialized nutritional counselling. However, its use should not replace the provision of healthy eating advice to individuals with SCI.

## Figures and Tables

**Table 1:** Characteristics of included study sample.

Characteristics (N = 51)	
Age (years), mean (SD)	57 (16.6)
Sex, number and percentage of males	39 (76.5%)
Injury etiology	
Traumatic, number (%)	35 (68.6%)
Non-traumatic, number (%)	16 (31.4%)
Injury level	
Cervical, number (%)	20 (39.3%)
Thoracic and lumbo-sacral, number (%)	31 (60.7%)
Injury completeness ^1^	
Motor complete injury (AIS A and B), number (%)	21 (41.2%)
Motor incomplete (AIS C, D and E), number (%)	30 (58.8%)
BMI (kg/m^2^), mean (SD)	25.2 (5.8)
Number (%) of individuals adherent to physical activity recommendation ^2^	12 (26.7%)
Comorbidities	
Diabetes type 1 and type 2, number (%)	2 (3.9%)
History of cardiovascular diseases, number (%)	1 (1.7%)
Obesity, number (%)	12 (23.5%)

Abbreviations: SD: standard deviation, BMI: body mass index. ^1^ Motor complete injury (AIS A or B), indicating no motor function preserved below the neurological level of injury; reported as number and percentage of participants; motor incomplete injury (AIS C, D, or E) indicating some preserved motor function below the neurological level of injury; reported as number and percentage of participants. ^2^ Adherence was defined as a minimum of 30 min of moderate-to-vigorous aerobic exercise performed three times weekly.

**Table 2:** Sensitivity and specificity of SCI NutriTool.

Food Groups	Number of Observations	Percentage of Individuals Non-Adherent to Dietary Recommendations	Sensitivity (Ability to Detect Non-Adherent Individuals)	Specificity (Ability to Detect Adherent Individuals)	Positive Predictive Value	Negative Predictive Value
Protein-rich foods ^1^	25	81.5%	90.5% (69.4–98.8)	50.0% (6.8–93.2)	90.5%	50%
Fruits and vegetables ^2^	33	72.7%	91.7% (73.0–98.9)	22.2% (2.8–60.0)	75.9%	50%
Starchy foods ^3^	33	15.2%	20.0% (0.5–71.6)	96.4% (81.7–99.9)	50%	87.1%
Nuts, oils and fatty spreads/sauces ^4^	32	6.3%	50% (12.6–98.7)	53.3% (34.3–71.7)	6.7%	94.1%
Sweetened, alcoholic beverages, and sweet and salty snacks ^5^	35	48.6%	82.4% (56.6–96.2)	72.2% (46.5–90.3)	73.7%	81.3%

***Criteria for acceptable adherence:*** *^1^ Acceptable adherence is defined as the consumption of no more than two servings of red and processed meat per week, along with one portion of protein-rich food (animal- or plant-based) per day and two to three portions of dairy products daily. ^2^ A daily intake of three or more portions of vegetables and two portions of fruits were considered acceptable. ^3^ Up to three daily portions of whole grains or starchy foods are acceptable. ^4^ One serving of unsalted, unprocessed nuts or seeds per day, along with no more than three servings of plant oils or butter used for food preparation, and up to one serving per day of fatty spreads, creams, or sauces. ^5^ Participants were classified as non-adherent unless they met all of the following criteria: alcohol consumption within acceptable limits of ≤3 servings per week and a combined intake of sugar-sweetened beverages, sweets/desserts, and salty/fatty snacks be-low the defined threshold of 6 servings per week.*

**Table 3:** Factors associated with non-adherence to different food groups.

	Number and Percentage of Individuals Non-Adherent to Specific Food Group				
Personal Characteristics	Protein-Rich Foods (*n* = 27)	Fruits and Vegetables (*n* = 33)	Starchy Foods (*n* = 33)	Nuts, Oils, +Fatty Spreads/Sauces (*n* = 32)	Sweetened, Alcoholic Beverages and Salty Snacks (*n* = 35)
**Male**	19 (90.5%)	**22 (84.6%)**	5 (19.2%)	2 (8.7%)	**17 (60.7%)**
**Female**	3 (50.0%)	**2 (28.6%)**	0 (0.0%)	0 (0.0%)	**0 (0%)**
**Cervical SCI**	13 (92.7%)	10 (71.4%)	2 (13.3%)	1 (6.25%)	9 (60.0%)
**Other SCI**	9 (69.2%)	14 (73.7%)	3 (16.7%)	1 (6.25%)	8 (40.0%)
**Obese**	15 (75.0%)	15 (68.2%)	2 (8.7%)	2 (8.33%)	12 (48.0%)
**Nonobese**	7 (100%)	9 (81.8%)	3 (30.0%)	0 (0%)	5 (50%)
**Physically active**	15 (83.3%)	16 (66.7%)	4 (16.7%)	1 (4.2%)	12 (46.1%)
**Physically inactive**	7 (77.8%)	8 (88.9%)	1 (11.1%)	1 (12.5%)	5 (55.6%)

Column percentages are shown, reflecting the proportion of non-adherent individuals within each category (e.g., sex, injury level, obesity status, or physical activity). For example, for protein-rich foods, 19 of 21 men (90.5%) and 3 of 6 women (50%) were non-adherent, indicating higher non-adherence among men. Fisher’s exact test was used to compare proportions between groups, and statistically significant differences are shown in bold.

**Table 4:** Test–retest reliability of the SCI NutriTool.

Food Group	Percent of Observed Agreement	PABAK ^1^	Agreement
Protein-rich foods	80.0%	0.60	Moderate
Fruits and vegetables	77.1%	0.54	Moderate
Starchy foods	82.9%	0.66	Good
Nuts, oils and fatty spreads/sauces	74.3%	0.48	Moderate
Sweetened, alcoholic beverages, and sweet and salty snacks	81.8%	0.64	Good

^1^ Prevalence-Adjusted, Bias-Adjusted Kappa (PABAK).

## Data Availability

Data is available from corresponding author upon reasonable request.

## Abbreviations

The following abbreviations are used in this manuscript:

SCI	Spinal Cord Injury
FFQ	Food Frequency Questionnaire

## References

[1] Scott J.M., Warburton D.E.R., Williams D., Whelan S., Krassioukov A. (2011). Challenges, concerns and common problems: Physiological consequences of spinal cord injury and microgravity. Spinal Cord.

[2] Hu X., Xu W., Ren Y., Wang Z., He X., Huang R., Ma B., Zhao J., Zhu R., Cheng L. (2023). Spinal cord injury: Molecular mechanisms and therapeutic interventions. Signal Transduct. Target. Ther..

[3] Tallqvist S., Kauppila A.-M., Vainionpää A., Koskinen E., Bergman P., Anttila H., Hämäläinen H., Täckman A., Kallinen M., Arokoski J. (2022). Prevalence of comorbidities and secondary health conditions among the Finnish population with spinal cord injury. Spinal Cord.

[4] Raguindin P.F., Itodo O.A., Eriks-Hoogland I., Muka T., Brach M., Stucki G., Stoyanov J., Glisic M. (2024). Does cardiometabolic risk profile differ among individuals with traumatic and non-traumatic spinal cord injury (SCI): The evidence from the multicenter SCI cohort in Switzerland (SwiSCI). Spinal Cord.

[5] Glisic M., Stoyanov J., Mueller G., Schubert M., Jordan X., Hund-Georgiadis M., Pannek J., Eriks-Hoogland I. (2024). Changes in Secondary Health Conditions Among Individuals With Spinal Cord Injury After Transition From Inpatient Rehabilitation to Community Living. Am. J. Phys. Med. Rehabil..

[6] Farkas G.J., Caldera L.J., Hodgkiss D.D., Mitchell J.R., Pelaez T.F., Cusnier M.A., Cole A.J., Daniel S.G., Farrow M.T., Gee C.M. (2025). Cardiometabolic Risk in Chronic Spinal Cord Injury: A Systematic Review with Meta-Analysis and Temporal and Geographical Trends. J. Clin. Med..

[7] Adriaansen J.J., Post M.W., de Groot S., van Asbeck F.W., Stolwijk-Swüste J.M., Tepper M., Lindeman E. (2013). Secondary health conditions in persons with spinal cord injury: A longitudinal study from one to five years post-discharge. J. Rehabil. Med..

[8] Rodgers K.A., Kigerl K.A., Schwab J.M., Popovich P.G. (2022). Immune dysfunction after spinal cord injury—A review of autonomic and neuroendocrine mechanisms. Curr. Opin. Pharmacol..

[9] Ronca E., Scheel-Sailer A., Koch H.G., Gemperli A. (2017). Health care utilization in persons with spinal cord injury: Part 2—Determinants, geographic variation and comparison with the general population. Spinal Cord.

[10] Gemperli A., Ronca E., Scheel-Sailer A., Koch H.G., Brach M., Trezzini B. (2017). Health care utilization in persons with spinal cord injury: Part 1-outpatient services. Spinal Cord.

[11] Bell N., Kidanie T., Cai B., Krause J.S. (2017). Geographic Variation in Outpatient Health Care Service Utilization After Spinal Cord Injury. Arch. Phys. Med. Rehabil..

[12] Buzzell A., Chamberlain J.D., Gmünder H.P., Hug K., Jordan X., Schubert M., Brinkhof M.W.G. (2019). Survival after non-traumatic spinal cord injury: Evidence from a population-based rehabilitation cohort in Switzerland. Spinal Cord.

[13] Buzzell A., Chamberlain J.D., Eriks-Hoogland I., Hug K., Jordan X., Schubert M., Zwahlen M., Brinkhof M.W.G. (2020). All-cause and cause-specific mortality following non-traumatic spinal cord injury: Evidence from a population-based cohort study in Switzerland. Spinal Cord.

[14] Majdan M., Plancikova D., Nemcovska E., Krajcovicova L., Brazinova A., Rusnak M. (2017). Mortality due to traumatic spinal cord injuries in Europe: A cross-sectional and pooled analysis of population-wide data from 22 countries. Scand. J. Trauma Resusc. Emerg. Med..

[15] Yu X., Pu H., Voss M. (2024). Overview of anti-inflammatory diets and their promising effects on non-communicable diseases. Br. J. Nutr..

[16] Felleiter P., Krebs J., Haeberli Y., Schmid W., Tesini S., Perret C. (2017). Post-traumatic changes in energy expenditure and body composition in patients with acute spinal cord injury. J. Rehabil. Med..

[17] Raguindin P.F., Bertolo A., Zeh R.M., Fränkl G., Itodo O.A., Capossela S., Bally L., Minder B., Brach M., Eriks-Hoogland I. (2021). Body Composition According to Spinal Cord Injury Level: A Systematic Review and Meta-Analysis. J. Clin. Med..

[18] Henke A.M., Billington Z.J., Gater D.R. (2022). Autonomic Dysfunction and Management after Spinal Cord Injury: A Narrative Review. J. Pers. Med..

[19] Stojic S., Eriks-Hoogland I., Gamba M., Valido E., Minder B., Chatelan A., Karagounis L.G., Ballesteros M., Díaz C., Brach M. (2023). Mapping of Dietary Interventions Beneficial in the Prevention of Secondary Health Conditions in Spinal Cord Injured Population: A Systematic Review. J. Nutr. Health Aging.

[20] Sabour H., Javidan A.N., Vafa M.R., Shidfar F., Nazari M., Saberi H., Rahimi A., Emami Razavi H. (2012). Calorie and macronutrients intake in people with spinal cord injuries: An analysis by sex and injury-related variables. Nutrition.

[21] Tomey K.M., Chen D.M., Wang X., Braunschweig C.L. (2005). Dietary intake and nutritional status of urban community-dwelling men with paraplegia. Arch. Phys. Med. Rehabil..

[22] Lieberman J., Goff D., Hammond F., Schreiner P., Norton H.J., Dulin M., Zhou X., Steffen L. (2014). Dietary intake and adherence to the 2010 Dietary Guidelines for Americans among individuals with chronic spinal cord injury: A pilot study. J. Spinal Cord Med..

[23] Aquilani R., Boschi F., Contardi A., Pistarini C., Achilli M.P., Fizzotti G., Moroni S., Catapano M., Verri M., Pastoris O. (2001). Energy expenditure and nutritional adequacy of rehabilitation paraplegics with asymptomatic bacteriuria and pressure sores. Spinal Cord.

[24] Perret C., Stoffel-Kurt N. (2011). Comparison of nutritional intake between individuals with acute and chronic spinal cord injury. J. Spinal Cord Med..

[25] Farkas G.J., Pitot M.A., Berg A.S., Gater D.R. (2019). Nutritional status in chronic spinal cord injury: A systematic review and meta-analysis. Spinal Cord.

[26] Eriks-Hoogland I., Jordan X., Baumberger M., Seijas V., Huber B., Michel F., Thietje R., Müller L. (2024). Recommendations for long-term follow-up care of secondary health conditions in spinal cord injury/disorder: A systematic review. Front. Rehabil. Sci..

[27] Shim J.S., Oh K., Kim H.C. (2014). Dietary assessment methods in epidemiologic studies. Epidemiol. Health.

[28] Segal-Isaacson C.J., Wylie-Rosett J., Gans K.M. (2004). Validation of a short dietary assessment questionnaire: The Rapid Eating and Activity Assessment for Participants short version (REAP-S). Diabetes Educ..

[29] Frankenfeld C.L., Patterson R.E., Horner N.K., Neuhouser M.L., Skor H.E., Kalhorn T.F., Howald W.N., Lampe J.W. (2003). Validation of a soy food-frequency questionnaire and evaluation of correlates of plasma isoflavone concentrations in postmenopausal women. Am. J. Clin. Nutr..

[30] Yaroch A.L., Tooze J., Thompson F.E., Blanck H.M., Thompson O.M., Colón-Ramos U., Shaikh A.R., McNutt S., Nebeling L.C. (2012). Evaluation of Three Short Dietary Instruments to Assess Fruit and Vegetable Intake: The National Cancer Institute’s Food Attitudes and Behaviors Survey. J. Acad. Nutr. Diet..

[31] Taylor A.J., Wong H., Wish K., Carrow J., Bell D., Bindeman J., Watkins T., Lehmann T., Bhattarai S., O’Malley P.G. (2003). Validation of the MEDFICTS dietary questionnaire: A clinical tool to assess adherence to American Heart Association dietary fat intake guidelines. Nutr. J..

[32] Abreu S., Costa C.D.S., Liz Martins M. (2025). Adaptation and Validation of the Nova-UPF Screener for the Assessment of Ultra-Processed Food Intake in Portuguese Adults. Nutrients.

[33] Wijnhoven H.A.H., Elstgeest L.E.M., de Vet H.C.W., Nicolaou M., Snijder M.B., Visser M. (2018). Development and validation of a short food questionnaire to screen for low protein intake in community-dwelling older adults: The Protein Screener 55+ (Pro^55+^). PLoS ONE.

[34] Kiesswetter E., Siebentritt H.M., Schoene D., Kob R., Freiberger E., Sieber C.C., Visser M., Wijnhoven H.A.H., Volkert D. (2023). Validation of the German version of the Protein Screener 55+. Eur. J. Clin. Nutr..

[35] Tangney C.C., Rasmussen H.E., Richards C., Li M., Appelhans B.M. (2019). Evaluation of a Brief Sodium Screener in Two Samples. Nutrients.

[36] Harnack L.J., Lytle L.A., Story M., Galuska D.A., Schmitz K., Jacobs D.R., Gao S. (2006). Reliability and validity of a brief questionnaire to assess calcium intake of middle-school-aged children. J. Am. Diet. Assoc..

[37] Głąbska D., Malowaniec E., Guzek D. (2017). Validity and Reproducibility of the Iodine Dietary Intake Questionnaire Assessment Conducted for Young Polish Women. Int. J. Environ. Res. Public Health.

[38] Farkas G.J., Sneij A., McMillan D.W., Tiozzo E., Nash M.S., Gater D.R. (2022). Energy expenditure and nutrient intake after spinal cord injury: A comprehensive review and practical recommendations. Br. J. Nutr..

[39] Bundesamt für Lebensmittelsicherheit und Veterinärwesen (2024). Chweizer Ernährungsempfehlungen für Erwachsene.

[40] Bernstein M., Huot I., Morabia A. (1995). Amélioration des performances d’un questionnaire alimentaire semi-quantitatif comparé à un rappel des 24 heures. Santé Publique.

[41] Li Z., Wang X., Du H., Liu W., Zhang C., Talifu Z., Xu X., Pan Y., Zhang J., Ke H. (2025). Unraveling Spinal Cord Injury Nutrition: Effects of Diet on the Host and Microbiome. Adv. Nutr..

[42] Farkas G.J., Gorgey A.S., Dolbow D.R., Berg A.S., Gater D.R. (2019). Caloric Intake Relative to Total Daily Energy Expenditure Using a Spinal Cord Injury-Specific Correction Factor: An Analysis by Level of Injury. Am. J. Phys. Med. Rehabil..

[43] Lee B.Y., Agarwal N., Corcoran L., Thoden W.R., Del Guercio L.R. (1985). Assessment of nutritional and metabolic status of paraplegics. J. Rehabil. Res. Dev..

[44] Farkas G.J., Gater D.R. (2018). Neurogenic obesity and systemic inflammation following spinal cord injury: A review. J. Spinal Cord Med..

[45] Martin Ginis K.A., van der Scheer J.W., Latimer-Cheung A.E., Barrow A., Bourne C., Carruthers P., Bernardi M., Ditor D.S., Gaudet S., de Groot S. (2018). Evidence-based scientific exercise guidelines for adults with spinal cord injury: An update and a new guideline. Spinal Cord.

[46] Masson L.F., McNeill G., Tomany J.O., Simpson J.A., Peace H.S., Wei L., Grubb D.A., Bolton-Smith C. (2003). Statistical approaches for assessing the relative validity of a food-frequency questionnaire: Use of correlation coefficients and the kappa statistic. Public Health Nutr..

[47] Haldemann M., Stojic S., Eriks-Hoogland I., Stoyanov J., Hund-Georgiadis M., Perret C., Glisic M. (2024). Exploring lifestyle components and associated factors in newly injured individuals with spinal cord injury. Spinal Cord.

[48] Bornstein M.H., Jager J., Putnick D.L. (2013). Sampling in Developmental Science: Situations, Shortcomings, Solutions, and Standards. Dev. Rev..

[49] Leeflang M.M.G., Bossuyt P.M.M., Irwig L. (2009). Diagnostic test accuracy may vary with prevalence: Implications for evidence-based diagnosis. J. Clin. Epidemiol..

[50] Naska A., Lagiou A., Lagiou P. (2017). Dietary assessment methods in epidemiological research: Current state of the art and future prospects. F1000Research.

